# Insect herbivory (*Choristoneura fumiferana*, Tortricidea) underlies tree population structure (*Picea glauca*, Pinaceae)

**DOI:** 10.1038/srep42273

**Published:** 2017-02-16

**Authors:** Geneviève J. Parent, Isabelle Giguère, Gaby Germanos, Mebarek Lamara, Éric Bauce, John J. MacKay

**Affiliations:** 1 Centre d’étude de la forêt, Département des sciences du bois et de la forêt, Université Laval, Québec, Qc, G1V 0A6, Canada; 2 Institut de biologie intégrative et des systèmes, Université Laval, Québec, Qc, G1V 0A6, Canada; 3 Department of Plant Sciences, University of Oxford, Oxford, OX1 3RB, UK

## Abstract

Variation in insect herbivory can lead to population structure in plant hosts as indicated by defence traits. In annual herbaceous, defence traits may vary between geographic areas but evidence of such patterns is lacking for long-lived species. This may result from the variety of selection pressures from herbivores, long distance gene flow, genome properties, and lack of research. We investigated the antagonistic interaction between white spruce (*Picea glauca*) and spruce budworm (SBW, *Choristoneura fumiferana*) the most devastating forest insect of eastern North America in common garden experiments. White spruces that are able to resist SBW attack were reported to accumulate the acetophenones piceol and pungenol constitutively in their foliage. We show that levels of these acetophenones and transcripts of the gene responsible for their release is highly heritable and that their accumulation is synchronized with the most devastating stage of SBW. Piceol and pungenol concentrations negatively correlate with rate of development in female SBW and follow a non-random geographic variation pattern that is partially explained by historical damage from SBW and temperature. Our results show that accumulation of acetophenones is an efficient resistance mechanism against SBW in white spruce and that insects can affect population structure of a long-lived plant.

Interactions between a host plant and its associated herbivores may vary considerably within a sympatric area^1^. Their interactions are affected by biotic and abiotic factors which in turn can influence the fitness of one or both of the interactors. Variations in plant-herbivore interactions can be characterized by measuring the frequency of heritable phenotypes or genotypes associated with defence on temporal or geographical scales^2^,^3^. The degree of variation in polymorphism across geographic scales usually reflects a mosaic of coevolution^4^,^5^. One example of this was described in *Arabidopsis thaliana* where geographic patterns were shown at a polymorphic defence locus. Different glucosinolates produced by different alleles at the locus and variable allelic frequency across its European distribution were linked to defence specificity against two species of aphid^2^.

In long-lived plants such as trees, defence against insects has not been linked to a specific resistance mechanism on geographic scales. In one study, resistant phenotypes against white pine weevil (*Pissodes strobi* Peck) in Sitka spruce (*Picea sitchensis* (Bong.) Carr) were highly abundant in an area of high insect damage^6^. In lodgepole pine (*Pinus concorta* Douglas ex Loudon), geographic variation in the level of disease or insect resistance was linked to introgression with jack pine (*Pinus banksiana* Lamb.)^7^. Local adaptation to insects has also been shown by using reciprocal transplant experiments^8^. Although these experiments allow to identify general resistance in trees and local adaptation against herbivores, they did not permit to delineate tree-herbivore interactions on temporal or geographical scales. A clear a link between a molecular mechanism in a forest tree, adaptation to a major biotic stressor and population processes is missing.

Recently, a resistance mechanism was identified in white spruce (*Picea glauca* (Moench) Voss) against spruce budworm (SBW), *Choristoneura fumiferana*, Clems^9^. Both are North American species and are largely sympatric. In the east of their sympatric distribution, resistant and non-resistant white spruces were previously characterized^9^,^10^,^11^ following a local SBW outbreak spanning nine years^10^. Differences in fitness were observed between resistant trees which had survived with little defoliation (0–20%) whereas non-resistant trees had either died or survived after severe defoliation (30–70%). Resistant trees accumulated the foliar aglycon acetophenones piceol and pungenol whereas non-resistant trees which only accumulated the glycosylated forms, picein and pungenin^9^,^11^. The beta-glucosidase PGβGLU-1 was shown to be responsible for the release of piceol and pungenol from the glycosylated substrates and was linked to SBW resistance. This novel resistance mechanism was foliage specific, constitutive, and variable in the eastern white spruce population; furthermore, the observed variation was under genetic control and genetically transmitted from parents to offspring was shown in selected genotypes^9^.

White spruce lives up to 150 years, has a large genome (20 GB^12^), and has two North American lineages with very weak population structure within each lineage. Eastern and western lineages derived from distinct glacial refugia were previously identified with neutral genetic markers^13^. Within the eastern lineage, weak population structure was observed^14^. An area located between the 46^th^ and the 49^th^ parallels north and the 64^th^ and 74^th^ west meridians, was severely affected by SBW during the last century^15^ in the eastern lineage.

The SBW is a lepidopteran native to North America east of the Rocky Mountains. It is a specialist herbivore whose larvae feed preferentially on current-year foliage and reproductive buds of fir (*Abies balsamea*, Mills.) and white spruce. No population structure has been detected across North America based on neutral genetic markers^16^. The eruptive population of SBW covers very large areas through the putative combined effect of climate, forest type, landscape, and natural enemies^17^,^18^. Its population density is highly variable and has reached maxima every 30 to 50 years over the last centuries^19^. After an epidemic, around half of the white spruce trees (49%) may die within a forest stand^20^.

Intraspecific variation in the acetophenone resistance mechanism against SBW may explain variability in insect fitness in white spruce. A previous study compared laboratory-reared SBW fed with piceol and pungenol and found reduced survival (51%) and slower development in females when both compounds were present at concentration as high as those observed in resistant trees^11^. These observations indicate that the compounds may increase tree fitness by reducing insect fitness as observed in the field^10^. However, a bioassay is necessary to confirm effects of aglycon acetophenones in white spruce.

The general aim of this research is to improve our understanding of interactions between trees and insect herbivores. In the present report, we pursued two broad objectives through investigation of the white spruce and SBW system. First, we studied the intensity of interactions by characterizing i) the phenology of both the tree and the insect in relation to acetophenone accumulation, and ii) the effect of foliar aglycon acetophenone concentrations on insect fitness *in situ*. Second, we characterized the factors affecting levels of resistance traits by i) estimating the effects of genetics and environmental factors, ii) identifying geographic variation of the host resistance mechanism, and iii) explaining patterns of geographic variation based on biotic and abiotic factors. Trees were characterized based on molecular and metabolite phenotypes associated with this constitutive and heritable resistance^9^. We use common garden experiments to compare phenotypes of many geographic origins under uniform environmental conditions and thus, compare the genetic proportion of phenotypic variance between origins^21^. With these data, we attempt to measure the effect of SBW on white spruce population structure based on these resistance phenotypes.

## Results

A total of 419 white spruce trees from three independent common gardens experiments (for details, see methods) were surveyed for the foliar accumulation of *Pgβglu-1* transcripts and acetophenone compounds. Individual common gardens were used to characterize the phenology of resistance traits (i.e. piceol, pungenol, transcripts of *Pgβglu-1*) in detail and measure the fitness of insects placed on trees with wide ranging concentrations of aglycon acetophenones, or estimate the heritability resistance trait by using molecular marker data. All three of the common gardens were used to characterize factors affecting phenotypic variability in resistance traits across the eastern lineage of white spruce.

### Tree and insect phenology

**Figure 1:**
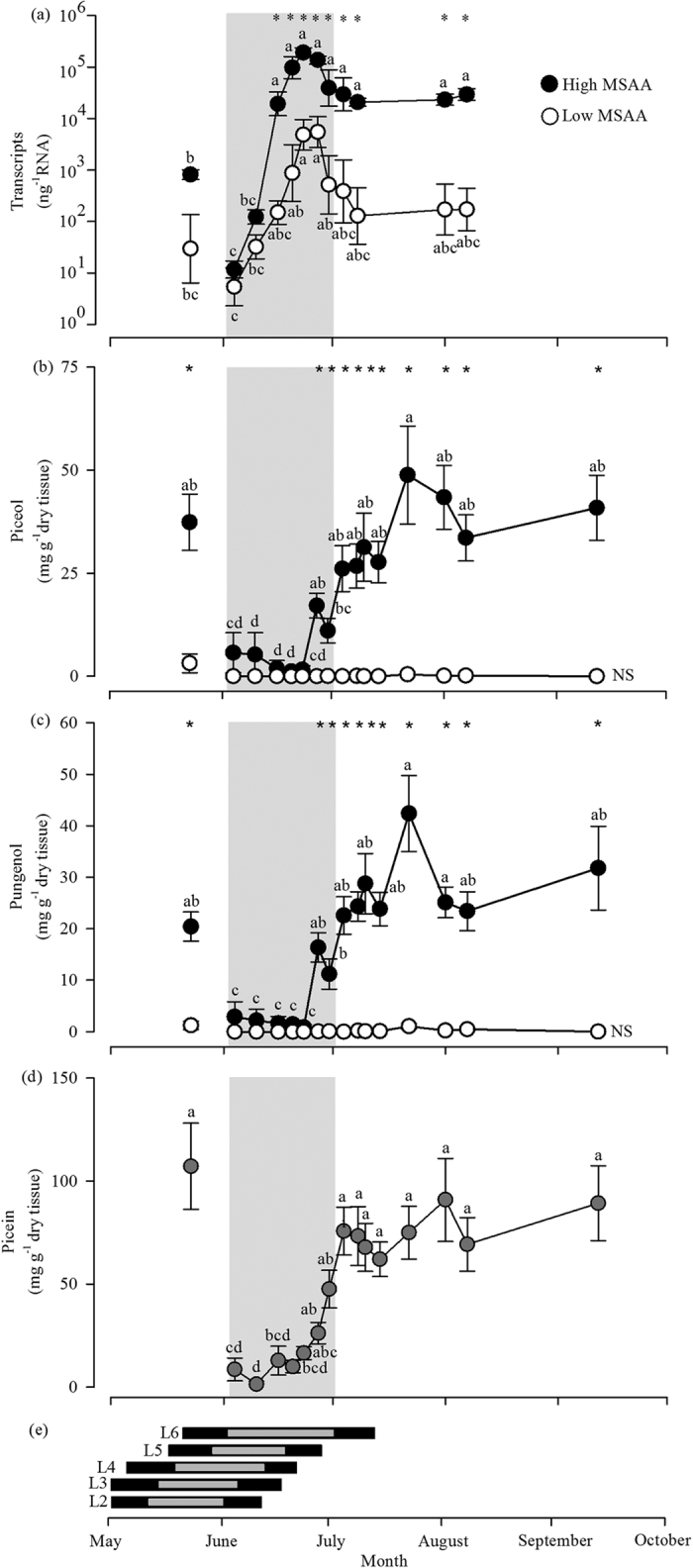
Phenology of white sprue (*Picea glauca*) and spruce budworm (SBW, *Choristoneura fumiferana*). (**a–d**) White spruce foliar phenotypes over the growth season of 2014. Mean traits for each class are presented for 12 and 16 sampling dates for transcript levels and acetophenone concentrations, respectively. For all classes *n* = 10, except for low mean sum aglycon acetophenones (MSAA) class for *PgBglu-1* transcripts where *n* = 5. The individuals in each profile class are the same for the four phenotypes. Grey circles represent the 20 individuals since no difference was observed between low and high classes of MSAA at any dates for picein. Stars and different letters indicate significant differences between classes and dates, respectively (analysis of variance type III testing for class, date, and their interactions effects; class*date: *PgBglu-1* transcripts *F*_*11*_ = 2.06 *P* = 0.03, piceol *F*_*15*_ = 9.27 *P* < 0.0001, pungenol *F*_*15*_ = 16.2 *P* < 0.0001; date: picein *F*_*15*_ = 11.4 *P* < 0.0001; Tukey multiple comparison test, α = 0.05). Mean and standard error are presented. The grey shading indicates the period of development of SBW instar 6 in 2014 based on modelling. (**e**) Time interval of SBW developmental stages. Black and grey bars indicate the time interval during which each insect stage is present based on simulations from 1965 to 2014 and in 2014, respectively, with BioSIM v10.3^40^. The beginning and end of the bars indicate minimum date of appearance of 1 and maximum date of 100% abundance of each developmental stage.

The accumulation of aglycon acetophenones was monitored in the current year foliage of 30 white spruce trees over the course of 2014 growing season. No temporal clustering patterns were observed for foliar content in piceol and pungenol (K-means, *n* = 1, principal component analysis presented in Supplementary Fig. S1). This was expected as the trees were initially selected to represent a wide range of concentrations for these specific acetophenones (see methods). Next we aimed to describe the extreme resistance profiles and decided to form two classes which were comprised of the ten individuals with the lowest or the highest mean sum of aglycon acetophenones (MSAA) across the time interval and compare the trees of the two MSAA classes for all of the measured traits (Fig. 1).

Early in the growth season, low levels were recorded for both *Pgβglu-1* transcripts and aglycon acetophenones in the current year foliage (Fig. 1). The *Pgβglu-1* transcripts began to increase, rapidly peaking and then decreasing in June in both of the MSAA classes (Fig. 1a); however, the high MSAA class trees reached higher *Pgβglu-1* transcripts levels during the June peak and remained two orders of magnitude higher for the rest of the sampling than in low MSAA class trees. The patterns for piceol and pungenol concentrations were similar but more accentuated as they increased only in the high MSAA class trees and accumulations were observed later, between mid-June and mid-July (Fig. 1b,c). In contrast, the foliar picein concentration profiles were similar in the two MSAA classes; they increased at the end of June and stayed high until the end of the sampling (Fig. 1d). For both of the MSAA classes, acetophenone concentrations in early July (10^th^) were similar to that observed later on in the season (August 7^th^, September 12^th^) and even in foliage formed in the previous year (May 23^rd^) (Fig. 1b–d). Throughout the time interval, *Pgβglu-1* transcripts were positively correlated with both of the aglycon acetophenones (Pearson’s correlation, *r*_piceol_ = 0.29, *P* < 0.0001; *r*_pungenol_ = 0.35, *P* < 0.0001), but not with the glycosylated acetophenone. Piceol is also positively correlated highly with pungenol (*r* = 0.74, *P* < 0.0001) and moderately with picein (*r* = 0.32, *P* < 0.0001).

We compared the timing of aglycon acetophenone accumulation and that of SBW development. The occurrence of SBW larvae stages L2 to L6 was investigated for an interval spanning the last 50 years and were found to be between April 17^th^ to July 11^th^ at the study site (Fig. 1e). Piceol and pungenol concentrations increased in the current year foliage near the third of the interval of L6 stage development which begins around May 20^th^ (Fig. 1b,c,e). In 2014 (the year of tissue sampling), L6 development was from June 2^nd^ to 30^th^ and ended when piceol and pungenol concentrations were 11.0 and 11.2 mg g^−1^ dry weight, respectively, in the high MSAA class trees and close to zero in the low MSAA class trees (Fig. 1b,c,e).

### Insect fitness with resistance traits of tree

In insect field trials, SBW females were affected by higher concentrations of aglycon acetophenones in white spruce foliage (Table 1). The development time of females was positively correlated with piceol and pungenol concentrations but there were no significant correlations for any of the variables for males (Table 1). We also tested the correlation between the sum of aglycon acetophenones to simplify our indicator of resistance and found that it was correlated with development duration of females but not males as observed for individual aglycon acetophenone (Table 1).

### Genetic and environment components of resistance traits

Traits associated with resistance were variable across common gardens. The mean concentrations of all of the acetophenones but not the *Pgβglu-1* transcript levels were lower in the Sussex common garden compared to the two other common gardens (see Supplementary Table S1). Concentrations of picein, piceol, and pungenol in Sussex were around 20%, 20 and 25%, respectively, of those from the Valcartier common garden. These differences could be due to genetic or environmental effects.

The accumulation of *Pgβglu-1* transcripts and aglycon acetophenones in current year foliage is mainly controlled by genetics. Estimates of heritability for *Pgβglu-*1, piceol and pungenol were of *h*_i_^2^ = 0.58, 0.67, 0.65, respectively. A relatively lower heritability estimate was obtained for picein (*h*_i_^2^ = 0.27). Thus, phenotypic variation in resistances traits (i.e. piceol, pungenol, transcripts of *Pgβglu-1*) is under strong genetic control.

We also compared resistance traits between the 19 origins that were common to Valcartier and Mastigouche common gardens to assess environmental effect with a different approach. Levels of all of the acetophenones, but not *Pgβglu-1* transcripts, were on average significantly higher in Valcartier (Table 2). These results indicate a significant effect of environment on resistance traits.

### Geographic variation of tree resistance

Origins with different selective pressure within each common garden could explain variation of mean acetophenones concentrations between common gardens. Thus, we compared biotic and abiotic factors in all origins in each common garden. Origins in Valcartier and Mastigouche common gardens have similar biotic and abiotic conditions compared to those of Sussex common garden (see Supplementary Fig. S2). Origins within the Sussex common garden are located further east in the white spruce distribution compared to the two other common gardens (see Supplementary Table S1) with higher mean annual precipitation and temperature, and are from lower altitudes than those in Valcartier and Mastigouche (see Supplementary Table S2).

**Figure 2:**
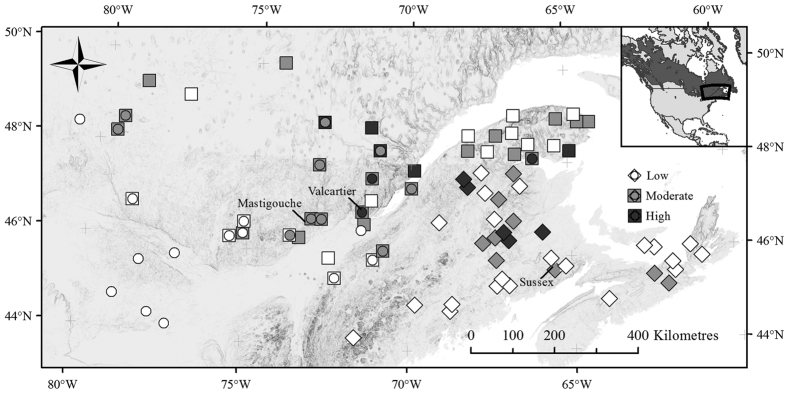
Geographic variability in white spruce (*Picea glauca*) resistance to spruce budworm (*Choristoneura fumiferana*) across their eastern distributions. In the insert, the black square represents the area of white spruce origins monitored for resistance traits from the white spruce distribution (dark grey). Circles, squares, and diamonds represent origins tested in Valcartier, Sussex, and Mastigouche common gardens, respectively. See Supplementary Table S3 for description of mean sum aglycon acetophenones (MSAA) classification within each common garden and material and methods, results for more details. Maps were created with ArcGIS v10.3 (ESRI, Redlands, CA, www.arcgis.com) and geospatial data (i.e. country limits and elevation) were obtained from GeoGratis.gc.ca.

Next, we present levels of resistance for origins in each common garden to show its the geographic variability (Fig. 2). In each common garden, origins were classified in one of the three levels of resistance determined automatically by the Jenk classification method in ArcGIS v10.3. We chose a method of automated classification to simplify the representation of MSAA levels and also to avoid any bias in classification that could result from a manual intervention (see methods for more details). Within each common garden, most of the origins were assigned to low or moderate MSAA classes (Fig. 2). In Valcartier and Sussex, the low MSAA were the most frequent (50 and 60%, respectively) whereas moderate MSAA levels were more prevalent in Mastigouche (50%, Fig. 2). Note that the 19 origins represented in two different common gardens were classified in identical (63%) or in the nearest class (37%) (Fig. 2) showing consistency in classification across common garden origins. These results also suggest that there is no genetic by environment (GxE) effect on resistance phenotype although it was not directly tested here. Across all of the origins tested in the three common gardens, the proportion of individuals (*n* = 359) with no aglycon acetophenones increased from the high (9%), to the moderate (12%) and to the low (30%) MSAA classes. These last results show that the classes using the automated approach (see methods) reflect reliable levels of resistance that are not driven by biased mean values of aglycon acetophenones used for origins.

We then tested each common garden for statistically significant spatial clusters of high and low MSAA values and detected four spatial clusters (Fig. S3). One cluster of low MSAA origins is located in the south west of the studied area and contains origins present in Valcartier common garden. Three further spatial clusters of high MSAA are located in the centre of the studied area from origins present in all three common gardens, between the 46^th^ and 49^th^ north parallels and the 66^th^ and 74^th^ west meridians (Fig. S3).

### Explanation of geographic variation in resistance

**Figure 3:**
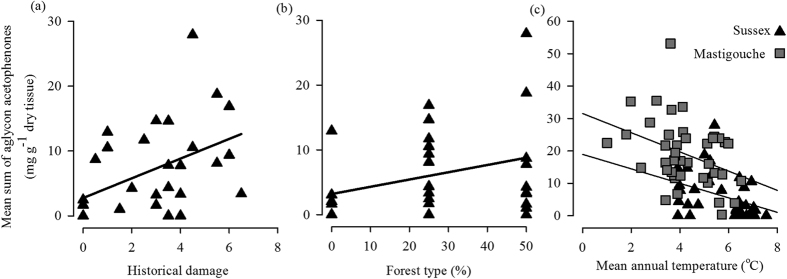
Explanation of geographic variation of resistance in white spruce (*Picea glauca*) within common gardens. Panels a to c show linear regression between mean sum of aglycon acetophenone and explanatory variables in common gardens. Only linear regressions with *P* < 0.05 from Supplementary Table S4 are presented.

Historical damage, forest type, and temperature partially explain the geographic variation in resistance traits (Fig. 3, see Supplementary Table S4). The predictive value of MSAA levels ranged from moderate (e.g. 0.26 historical damage in Sussex) to low (e.g. 0.09 mean annual temperature in Mastigouche, see Supplementary Table S4). Historical damage and forest type are positively related to MSAA only in Sussex common garden (Fig. 3). Mean annual temperature is negatively correlated to MSAA and is a significant predictive variable in Mastigouche and Sussex common gardens, and is almost significant in Valcartier (see Supplementary Table S4).

Exploratory regressions indicated that historical damage and mean annual temperature explain part of the variation in resistance observed in the whole study area. In the best model, *r*^2^ adjusted was of 0.22 and was explained positively by historical damage (log transformed) and negatively by mean annual temperature the three classes of MSAA in the 84 origins across the eastern distribution. This model passed all of the criteria stated in the method with default parameters of the analyses, except for the test for random distribution of model residuals. This indicates that at least one explanatory variable is missing in the model.

## Discussion

This study examined the interactions between a tree and an insect. It provides insights into the temporality and effect of their interactions on the white spruce population structure. Our findings on phenology and impacts on insect fitness combined with those from laboratory-based bioassays^11^ clearly indicate that the accumulation of piceol and pungenol is an efficient and predictable defence mechanism against SBW in white spruce. We show that it reduces the fitness in SBW females by slowing their development and explains the improved fitness of resistant trees in naturally-occurring epidemic conditions^10^. Historical damage and temperature explained variation in the resistance traits observed across a large area of the distribution white spruce. Although numerous studies have shown resistance mechanisms against insects or pathogens in trees^22^, few attempts have been made to characterise the antagonistic interactions on temporal or geographic scales to provide greater insights into the evolution of defence in long-lived plant species^7^.

We show that the peak of piceol and pungenol accumulation in current year foliage observed in several trees temporally matches SBW larval stage that is the most damaging. The results were obtained from trees that were not infested by SBW at the time of sampling and no prior exposure to SBW had been documented, which indicates that the mechanism is constitutive as previously suggested^9^. The MSAA concentration increased in current year foliage at the end of June during larval stage 6, which accounts for 87% of consumption by all stages^23^. A sharp increase was characteristic of high MSAA trees and was not observed in trees with very low MSAA. Here we show that the phenotypic contrast as previously reported^9^ was maintained during the remainder of the growth season. The MSAA resistance phenotype was consistent from year to year as seen by comparing foliage from the previous year and the end of season in the current year foliage (Fig. 1). This consistency could explain why the trees with low concentration of piceol and pungenol were more severely damaged throughout by SBW during a local outbreak^10^. The outbreak lasted nine years and high MSAA (resistant) trees had lower defoliation and better survival^10^,^11^. Taken together, insect and tree phenology results show that the resistance mechanism matches the development of SBW in some white spruce trees and can act as an efficient constitutive defence against SBW.

Development of females SBW was slower in trees that reached higher concentrations of piceol and pungenol. This result is consistent with those obtained with SBW fed on artificial diet supplemented with the pure compounds^11^. In our field insect trial, MSAA concentration in current year foliage reached concentrations equal or greater than those tested in artificial diet for 10 out of the 29 trees (i.e. piceol > 3.37 mg g^−1^ and pungenol > 2.70 mg g^−^). The end of June MSAA concentrations were low compared the end of the season levels but were sufficient to have an impact on the development of female insect. A reduced developmental rate of SBW can decrease survival through higher risk of predation among others^24^ and could be amplified by a negative feedback as increasing MSAA are observed during foliage development. Unlike Delvas and coll.^11^ which used an artificial diet to test the effect of piceol and pungenol, we did not observe a reduction in survival which we mainly attributed to differences in experimental set up including small sample sizes and insect escapes from the mesh bags during set up. Alternatively, other compounds in the foliage may have obscured the impact of piceol and pungenol on survival.

The degree of synchrony of spruce trees and SBW phenology may increase or decrease the effectiveness of the resistance mechanism in white spruce. In 2014, the increase in MSAA levels occurred over a short period near the end of stage L6 in the insect development model and the field insect trial. If the insects develop earlier, the resistance mechanism may not be effective and the phenological window that is favourable for insect herbivory may become wider. An example of synchronized phenology between an insect and its tree host comes from the study of gall forming insects and oaks where phenological matching is the key to susceptibility^25^. In our study, we did not report on the interannual variation of tree phenology which we are presently investigating.

On the geographical scale, we showed that variation of these SBW resistance traits across the eastern distribution of white spruce is not random. The three common gardens show similarity in the geographic variation of resistant and non-resistant trees. The results are not clear-cut (i.e. only origins with high levels of MSAA in an area) but a clear trend in the distribution of resistant and non-resistant trees is observed and supported statistically. This population structure in resistance traits is also supported by our ability to explain its geographic variation. Historical damage and mean annual temperature explain as much as 26% of the geographic variation of resistance in the whole study area. This result supports that there is population structure and that SBW likely affects it. It also shows that geographic variation in resistance associated with an insect can be detected in trees based on resistance traits.

High levels of historical SBW damage were associated with origins with high MSAA trees within one common garden (i.e. Sussex) and across the studied area. It seems rather intuitive that resistant trees are more abundant where the selective pressure was greater; however, quantitative explanations of non-random distribution for resistance are scarce in trees^7^,^26^. The availability of quantitative data of historical SBW damage standardized over the Canadian distribution of white spruce^15^ allowed us to use a quantitative approach to test for the effect of SBW on population structure of white spruce.

Temperature was also an explanatory variable of geographic resistance. It is well known that temperature affects the survival of the species studied here, which are both poikilotherms. Temperature largely determines the rate of physiological processes and affects the distribution of both spruce and SBW^27^. For the trees, the resistance phenotypes were measured in common garden experiments; therefore, most of the variation is associated with standing genetic variation or local adaptation seeing as no population structure based on neutral markers underlies this resistance structure. We also showed that the environment had a significant effect on phenotype; this is not surprising seeing as the temperature has a large effect on general^28^ or resistance phenotypes^29^,^30^. In fact, it has been shown that piceol and picein concentrations in Norway spruce (*P. abies* (L.) Karst) increase with decreasing temperatures, which may indicate a role in cold acclimation^29^ but this issue is beyond the scope of the present study. For SBW, temperature is an important factor affecting development and survival across the landscape^31^. It has been hypothesized that temperature observed in the south is less favourable for SBW survival compared to that of the northern part of our study area^31^, which could contribute to lowering the selection pressure exerted by SBW on white spruce in this area.

Interactions between the effect of historical SBW and temperature damage may also affect our ability to explain geographic variation across this large studied area. The temperature range covered in Mastigouche and Valcartier is wider than that of Sussex (see Supplementary Table S2, Fig. 3c). In addition, mean annual temperatures are lower in origins from Mastigouche and Valcartier (see Supplementary Table S2). Thus, the selection from the grazing pressure by SBW may be easier to detect in origins from the Sussex common garden because the effect of temperature is smaller.

Other factors could explain the geographic pattern we observed for MSAA. We identified forest type as a factor that positively explains the mosaic geographic variation in resistance in one common garden. It has been shown that the presence or abundance of hardwood trees have a negative impact on defoliation by SBW which could be associated with the presence of more abundant natural enemies^32^. Still other factors could explain phenotypic resistance variance at the geographic scale since most phenotypic variation (*ca.* 74%) could not be explained by the five independent variables we were able to test.

Our results combined with findings from previous studies on tree-insect interactions show support for local adaptation to SBW in white spruce. Previous studies have shown that SBW has a large impact on white spruce fitness^20^, and that there are differences in fitness in the natural population^10^. In this study, we show how the phenology of SBW development match that of the defence mechanism in resistant white spruce. We also show a lower fitness of SBW on resistant trees with a bioassay. These results describe the antagonistic interactions between white spruce and SBW and explain the difference in survival across white spruces reported in previous studies^10^. We also estimated that around 60% of the phenotypic variance in resistance traits (i.e. piceol, pungenol, transcripts of *Pgβglu-1*) is under genetic control which allow possible evolution of these traits in the natural population. These different lines of evidences clearly indicate that the non-random geographic variation in resistance traits is explained at least in part by local adaptation rather than standing variation alone.

The geographic mosaic of resistance in white spruce likely reflects the combined effects of the resistance mechanism studied here and the recurring defoliation by the endogenous SBW over the last four hundred years^19^ and potentially across millennia^33^. We observed a population structure associated with resistance in white spruce, although the level of gene flow reported within the eastern lineage^14^ may be expected to erase geographic patterns. This pattern suggests that the fitness of white spruces with low and high MSAA varies across the study area. We believe that the difference in fitness between low and high MSAA trees may be small and that this would be due to four main factors. First, the survival of low MSAA trees may be unaffected during some epidemics due to mismatches between SBW and white spruce resistance phenology. Second, the interval of time between epidemics is long enough for low MSAA trees to reproduce. Maturity in white spruce is at *ca*. 30 years and intervals between periodical epidemics is often greater than 30 years^19^. Third, the effects of SBW on white spruce are patchy within their sympatric distribution^34^. In areas unaffected by SBW, the fitness differential between low and high MSAA trees may be null, assuming that there is no allocation cost for defence. Fourth, non-resistant trees may survive following a light intensity epidemic as observed previously^10^. In this case, the cost of herbivory may not be large enough to reduce the fitness of non-resistant trees. Taken together, differences in fitness between low and high MSAA white spruce may be small but nonetheless it is likely that the recurrent selection pressure over the last hundreds or thousand years caused the geographic pattern we observed in our study area.

## Material and Methods

### Sampling

#### Phenology and fitness

Tree phenology of resistance traits: Foliage was sampled at 16 dates (May 23; June 4, 10, 16, 20, 23, 27, 30; July 4, 8, 10, 14, 22; August 1, 7, and September 12) in 2014 from 30 mature white spruces (*Picea glauca*) selected for their diversified origins and overall variability in piceol and pungenol content based on results presented in Mageroy and coll^9^. These trees were located in a common garden established in 1999 in Valcartier, Quebec, Canada (46°56′N, 71°29′W).

#### Insect fitness related to tree resistance traits

Thirty other white spruces from Valcartier common garden were selected for their overall variability in piceol and pungenol content based on results presented in Mageroy and coll^9^. for an insect field rearing experiment from May to July 2014. During this period, foliage was sampled from trees on June 6, 10, 23, and July 10. Two fine mesh bags of 1 m in length and with 20 post-diapausing second-instar larvae (Canadian Forest Service, Sault Ste. Marie) of spruce budworm (*Choristoneura fumiferana*) inside of them were added on branches of each tree on May 29. Development of insects was monitored every three days. On July 2, branches were transferred to the laboratory to determine sex and to measure mass of pupae. Larvae that were not at pupal stage were reared on an artificial diet^35^ until they reach this stage. Moth emergence was monitored every hour to determine developmental time from L2 stage to adult. Survival was estimated after insects had reached the adult stage and was based on the number of moths that emerged per bag.

#### Factors affecting phenotypes of resistance traits

Foliage was sampled from mature *Picea glauca* trees from 144 different origins covering a large geographic area (maximal length of 1530 km) in the species’ Eastern range. The trees were from three common garden experiments: (1) Valcartier (same as in Mageroy *et al*.^9^, sampled on October 1st 2013), (2) Mastigouche, Quebec, Canada (46°38′N, 73°13′W) established in 1979 (described in Beaulieu *et al*.^36^) sampled on July 23 and 24 2014, and Sussex, New Brunswick, Canada (45°44′N, 68°29′W, JD Irving Company) established from 1988 to 1992 and sampled August 12 2014. In the Valcartier common garden, 41 trees from 26 origins (*n* = 1 to 5 trees, mean = 2 trees per origin) were sampled in 2013 (see population tree from Mageroy *et al*.^9^). In 2014, 214 trees from 42 origins (*n* = 4 to 6, mean = 5) and 107 trees from 95 origins (*n* = 1 to 3, mean = 1) were sampled from Mastigouche and Sussex, respectively. In total, 19 origins were common to the Valcartier and Mastigouche common gardens.

#### Sampling details and sample preparation

For all of the trees, current-year foliage was sampled in the midcrown from the north side of the tree. The samples were frozen immediately in liquid nitrogen after removal from the trees and stored at −80 °C. Foliage was ground to a fine powder using a MixerMill 300 (Retsch) and steel grinding balls cooled in liquid nitrogen. Powdered tissue was stored at −80 °C until further analyses. Samples were assessed for *PgBglu-1* transcripts and acetophenones, which are also identified as resistant traits^9^.

## Laboratory analyses

### RNA extraction and transcripts assay

Total RNA was extracted as in Chang *et al*.^37^ with modifications as in Pavy *et al*.^38^ and stored at −80 °C. The total RNA concentration was determined using a NanoDrop 1000 (Thermo Scientific) and assessed for quality with an Agilent 2100 Bioanalyzer and RNA 6000 Nano Kit LabChips (Agilent Technologies Inc.).

Reverse transcriptase-qPCR with gene-specific primers was used to quantify *PgBglu-1* transcripts (see Mageroy *et al*.^9^ for more details). Complementary DNA synthesis used 500 ng of total RNA and the Superscript First-Strand cDNA synthesis system for RT-PCR (Invitrogen). The PCR mixtures were composed with a QuantiFast SYBR Green PCR kit (Qiagen) as follows: 1x master mix, 300 nM of 50 and 30 primers and 5 μl of cDNA in a final volume of 15 μl. Amplifications were carried out in a LightCycler 480 (Roche) as described in Boyle *et al*.^39^ and the LRE method^40^ was used to calculate the number of transcript molecules.

### Acetophenone extraction and assay

Acetophenones were extracted as described in Mageroy and coll.^9^. Assays were conducted on a LC (Agilent 1200 series) coupled to a MS detector (Agilent 6210 TOF). This differs from our previous publication^9^ as we detected interference with other compounds that had not been accounted for properly. Previously published findings remain unchanged since resistant and non-resistant trees have clearly different levels in aglycon acetophenones (see Supplementary Fig. S3); however, the quantitative determinations of aglycon acetophenones in current year foliage were lower than reported in resistant trees and not detected in several non-resistant trees with the MS assay compared to previous UV assay results^9^. Quantification with MS and UV are moderately to highly correlated for population data from Fig. 6 in Mageroy and coll.^9^ (*n* = 39, Pearson’s correlation, *r*_piceol_ = 0.48, *P* = 0.002; *r*_pungenol_ = 0.43, *P* = 0.006; *r*_picein_ = 0.83, *P* < 0.0001). Acetophenones were separated in a pre-column Polaris MetaGuard 4.6 mm and a column Polaris 250 mm 9 4.6 mm C18-A (Agilent Technologies Inc.). The solvent and solvent gradient were as described in Mageroy *et al*.^9^. The column flow rate was 1.5 ml min^−1^. Five microlitres of extract was injected. Quantification was done using calibration curves for picein, piceol and pungenol. No pungenin is commercially available.

### Models and statistical analyses

Principal component analysis (and K-means), correlations, regressions, paired t-tests, and analyses of variance (ANOVAs) were conducted with SAS 9.4 (SAS Institute Inc).

#### Phenology and fitness

Insect phenology simulations: SBW phenology model was run in BioSIM^41^ using average daily temperatures from the nearest 8 weather stations (Canada-USA), weighted according to the inverse of distance from target sites. This model uses temperature as the main factor influencing development. Simulations were done from 1965 to 2014.

#### Genetic and environmental effects on resistance traits

Estimates of narrow sense heritability: We used phenotypes obtained in this study and genotypes of 211 individuals (from the same number of families) from Mastigouche common garden that have been previously described^36^,^42^,^43^. A subset of 4767 single nucleotide polymorphism (SNP) markers (minor allele frequency ≥ 0.1) of high quality in 2312 genes distributed across the genome were used to produce a genetic kinship matrix (K) with Tassel v5 software^44^. A mixed linear model implemented in the R package “heritability”^45^,^46^ was then used to estimate narrow sense heritability.

#### Geographic variation of tree resistance

Clustering analyses of origins from Sussex common garden: Trees in Sussex common garden originate from 95 sites, with *n* = 1 for most origins. In Valcartier and Mastigouche common gardens, with *n* = 2 to 6 per origin where each individual came from different mother trees interspaced by *ca.* 1 kilometre. Thus, we grouped individuals originating within 10 km of each other from Sussex to form 35 “new origins” (*n* = 1 to 14 trees per origin, mean = 3 trees) and have a similar structure of origins across common gardens. This procedure was done using a buffer analysis with ArcGIS v10.3 (ESRI, Redlands, CA).

#### Biotic and abiotic conditions in origins

Five independent variables were considered to characterize conditions in each of the origins. Historical damage over the eastern distribution was obtained from Gray & MacKinnon^15^. We converted the graphical colour scale to a numerical scale from 0 to 7 to represent increasing levels of damage over the last century (i.e. 0 = absence to 7 = very severe damage) standardized over the eastern provinces of Canada. Forest type was estimated based on a 10 km radius from each origin from land cover map of Canada with 250 m resolution (2005 v2, Canada Centre for Remote Sensing) with ArcGIS v10.3. We classified forest type based on its minimum coniferous forest content (i.e. 0, 25, 50, 75, and 100%). Note that values for historical damage and forest type are missing for four origins in the United States (Sussex common garden). Altitude was extracted from a 10 km radius around each origin from an elevation map of North America with 1 km resolution (2007, Commission for Environmental Cooperation) using ArcGIS v10.3. Annual temperature and precipitation were simulated between 1965 and 2014, and then averaged for the 84 origins with the daily climatic model in BioSIM^41^.

#### Analyses to identify geographic patterns of resistance

We first used a classification approach in ArcGIS v10.3 to simplify visualization of geographic variation in resistance and to compare resistance patterns between common gardens. Each common garden was analysed separately as environment likely affect traits associated to resistance (see results). We then used hotspot analyses (Getis-Ord Gi*) in ArcGIS 10.3 v (default parameters) to test for significant aggregation of high and low values of MSAA in each common garden. In this analysis, an origin can be identified as a hot or a cold spot if it is surrounded principally by high or low values, respectively. For this test, we used MSAA values for each origin. Statistically significant clusters of origins identified as low or high MSAA are presented after correction for false discovery rate (FDR). Critical p-values determining confidence levels is reduced to account for multiple testing and spatial dependence when using FDR correction. We denoted an artefact to this analysis where origins on the outside of the distribution tend not to identify hot or cold spots even for data simulated as such. We believed that this could be due to the unequal distribution of origins across the landscape based on other simulations. Note that our low number of origins in each analysis may also contribute to this effect.

#### Explanation of geographic patterns of resistance

We first used linear regression to characterize the relation between variables (see biotic and abiotic conditions in origins) and MSAA of the origins within each common garden. We then used exploratory regression in ArcGIS v10.3 to identify variables explaining geographic variation in resistance across the 84 origins. Explanatory regression in ArcGIS v10.3, comparable to a step-wise regression, evaluates possible variable combinations (i.e. one to five variables) that form significant ordinary least squares (OLS) regression model. To maximize the utilization of our phenotyping across all three common gardens, classes of MSAA were used. For origins present in two common gardens, we averaged the classification and considered rounding up or down (7 out of 19 origins) to reduce the number of classes. Results were similar for both set of values and thus, we considered only rounding up.

## Additional Information

**How to cite this article:** Parent, G. J. *et al*. Insect herbivory (*Choristoneura fumiferana*, Tortricidea) underlies tree population structure (*Picea glauca*, Pinaceae). *Sci. Rep.* **7**, 42273; doi: 10.1038/srep42273 (2017).

**Publisher's note:** Springer Nature remains neutral with regard to jurisdictional claims in published maps and institutional affiliations.

## Supplementary Material

Supplementary Information

## Figures and Tables

**Table 1:** *In situ* characterisation of the relation between aglycon acetophenone concentrations in current year foliage of white spruce (*Picea glauca*) and fitness components of spruce budworm (*Choristoneura fumiferana*).

Tree	Insect				Survival
	Developmental duration		Pupal mass		
	Females	Males	Females	Males	
Piceol	0.66 (0.0001)	0.21 (0.28)	−0.25 (0.21)	−0.17 (0.39)	0.00 (0.99)
Pungenol	0.72 (<0.0001)	0.30 (0.11)	−0.28 (0.16)	−0.17 (0.39)	−0.03 (0.89)
Aglycon acetophenones (sum)	0.71 (<0.0001)	0.25 (0.19)	−0.27 (0.18)	−0.17 (0.37)	−0.01 (0.96)

Aglycon acetophenone concentrations represent an average of four samples across insect rearing experiment for each tree (*n* = 30 trees). Fitness components of insects are based on a mean of the two replicates (i.e. bag, *n* = 20 insects per bag) per tree. Coefficient and *P*-value (in parenthesis) of Pearson’s correlation are presented.

**Table 2:** Comparison of resistance traits in white spruce (*Picea glauca*) for origins present in both of the Valcartier and Mastigouche common gardens.

Common gardens	*Pgβglu-1* transcripts (log_10_ ng^−1^RNA)	Picein (mg g^−1^)	Piceol (mg g^−1^)	Pungenol (mg g^−1^)
Valcartier	4.4 ± 0.2	173.5 ± 21.7^a^	26.2 ± 5.6^a^	11.9 ± 3.0^a^
Mastigouche	3.3 ± 0.2	71.6 ± 9.6^b^	12.7 ± 2.0^b^	6.4 ± 0.8^b^

Different letters indicate significant differences with a paired student t-test between the two common gardens (*n* = 19, *df* = 18, *P* < 0.001).
