# Supplementary material for: Insect herbivory (Choristoneura fumiferana, Tortricidea) underlies tree population structure (Picea glauca, Pinaceae)
Source: Sci Rep. 2017 Feb 16;7:42273. doi: 10.1038/srep42273 (PMC5311968; doi:10.1038/srep42273)
Supplement: Supplementary Information [file srep42273-s1.pdf]

Supporting Information

Insect herbivory (*Choristoneura fumiferana*, Tortricidea) underlies tree population structure (*Picea glauca*, Pinaceae)

Geneviève J. Parent[1,2,3], Isabelle Giguère[1,2], Gaby Germanos[1,2], Mebarek Lamara[1,2], Éric Bauce[1], John J. MacKay [1,2,3]

[1]Centre d'étude de la forêt, Département des sciences du bois et de la forêt, Université Laval, Québec, Qc, Canada G1V 0A6 [2]Institut de biologie intégrative et des systèmes, Université Laval, Québec, Qc, Canada G1V 0A6 [3]Department of Plant Sciences, University of Oxford, Oxford, UK OX1 3RB

Author for correspondence:

Genevieve Parent

Tel: +44 070507 418 351

Email: genevieve.parent@plants.ox.ac.uk

Figures

Fig. S1

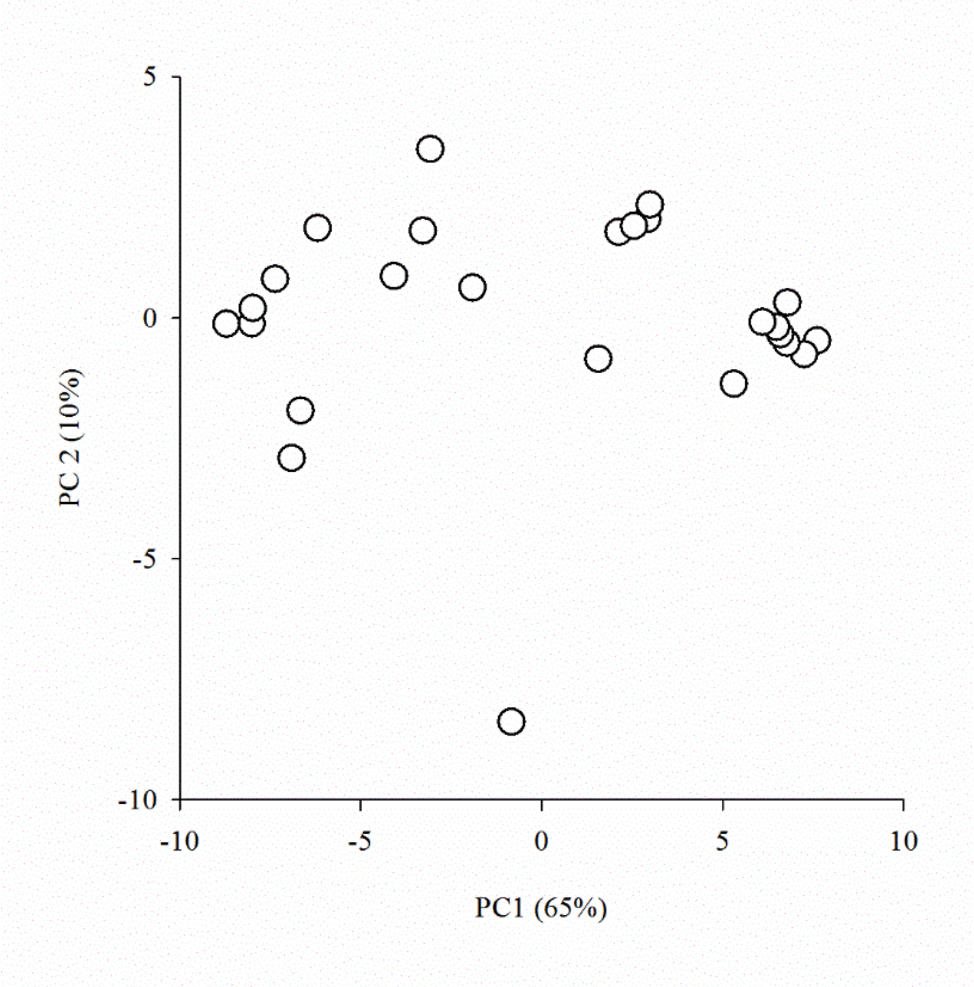

Variability in aglycon acetophenone concentrations in foliage of *Picea glauca* across the sampling interval. Principal component (PC) analysis using as variables concentrations in piceol and pungenol in current year foliage from the 16 samplings across the time interval for 30 mature white spruces. Each point represents an individual tree. Mean values for the date were used for missing data. A single cluster was identified in this PCA analysis (K-means model) showing a gradient of phenotypes and not classes of aglycon acetophenone content in foliage.

Fig. S2

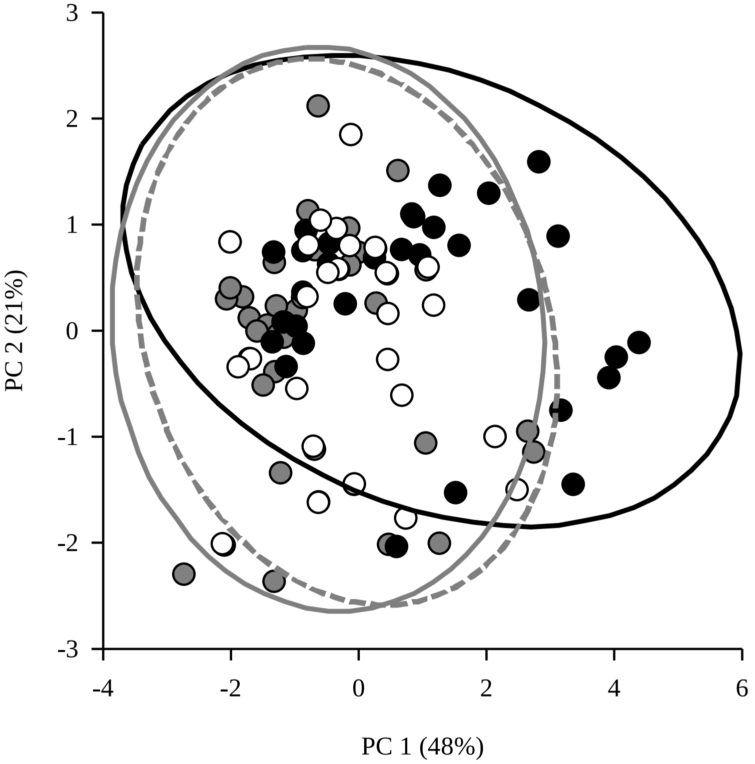

Variability in biotic and abiotic conditions in origins from the three common gardens. Principal component (PC) analysis using historical damage, forest type, altitude, temperature, and precipitation annual mean for 103 origins (19 origins common to Valcartier and Mastigouche common gardens). Grey dotted (Valcartier), grey (Mastigouche), and black (Sussex) continuous ellipses indicate the area covered by 95% of the origins in each common garden. Each dot represents an origin.

Fig. S3

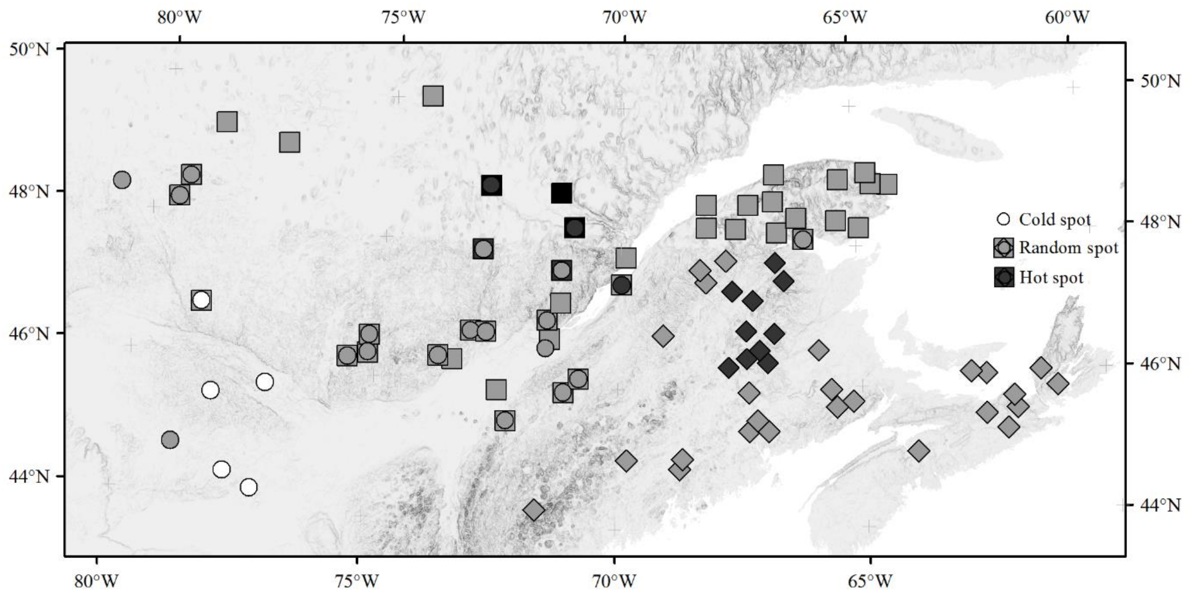

Spatial statistical analysis testing for clustering of high and low MSAA concentration in foliage of white spruces. The three classes presented are the bin issued from hotspot analyses (Getis-Ord Gi*) in ArcGIS 10.3v (default parameters). Hot or cold spots indicate significant clusters of origins with high or low MSAA concentrations, respectively. Random spot indicates no specific cluster of high or low MSAA concentrations surrounding that origin. Note that simulations showed that high MSAA origins simulated as such in the north west distribution were not detected as significant spatial clusters (see methods). The map was created with ArcGIS v10.3 (ESRI, Redlands, CA, arcgis.com) and geospatial data (i.e. country limits and elevation) were obtained from GeoGratis.gc.ca.

Fig. S4

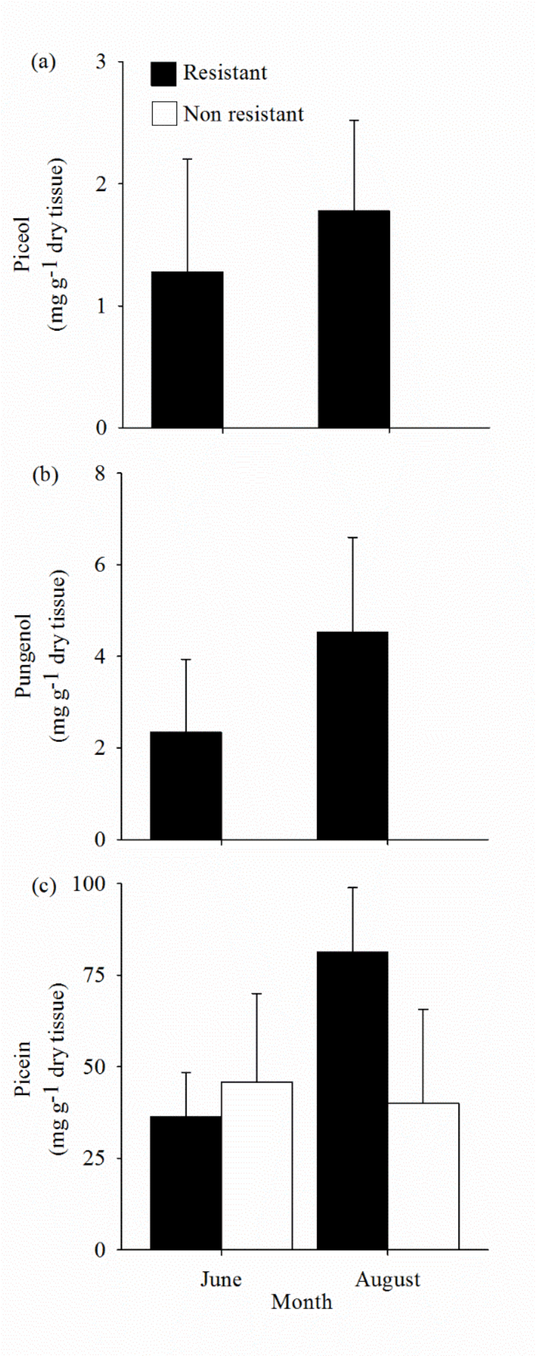

Acetophenone concentrations in the two resistance classes of white spruce (*Picea glauca*) quantified with mass spectrometry. Trees were sampled on 18 June and 13 August in 2013. An analysis of variance type III was used to test for month and resistance phenotype effects and their interactions. Only resistance phenotype effect was significant for piceol and pungenol (piceol $F_{1,18} = 6.22$, $P = 0.02$; pungenol $F_{1,18} = 6.94$, $P = 0.02$). Mean and standard error are presented.

Tables

Table S1. Characteristics of common gardens and variability in resistance traits in white spruce (*Picea glauca*). Minimum and maximum latitudinal (lat) and longitudinal (long) coordinates of the origins are indicated in the geographic coordinates. For acetophenones and transcript concentrations, minimum, mean, standard error, and maximum values for origins are presented in this order within each common garden. An analysis of variance type IV (unbalanced design) was used to test for common garden effect. Transcripts, picein, piceol, and pungenol concentrations were log transformed for proper residual normality. A star indicates significant difference across common gardens for the marked variable.

Common gardens	Origins	Geographic coordinates				*Pgβglu-1* transcripts*					Picein*					Piceol*					Pungenol*				
		Lat (º)		Long (º)		(log10 ng-1RNA)					(mg g-1)					(mg g-1)					(mg g-1)				
Valcartier	26	44.23	48.83	-80.75	-66.12	0.9	3.9	±	0.2	5.4	0.0	173.2	±	13.4	409.4	0.0	19.7	±	3.0	83.5	0.0	8.9	±	1.4	43.8
Sussex	35	44.25	47.83	-71.61	-61.02	0.7	3.1	±	0.0	4.5	0.0	33.5	±	1.2	183.5	0.0	3.8	±	0.2	16.6	0.0	2.2	±	0.1	11.3
Mastigouche	42	45.50	50.05	-79.48	-64.25	1.9	3.5	±	0.1	4.3	18.5	62.8	±	3.1	202.5	0.0	12.8	±	0.8	37.5	0.3	6.3	±	0.4	15.8

Table S2. Biotic and abiotic factors affecting the origins of the three common gardens. For all explanatory variables, minimum, mean, standard error, and maximum values for origins within each common garden are presented. An analysis of variance type IV (unbalanced design) was used to test for common garden effect. All variables except altitude were log transformed. A star indicates significant difference across common gardens for this variable.

Common gardens	Historical damage (0 to 7)					Forest type (% coniferous)					Altitude* (m)					Mean annual* temperature (°C)					Mean annual rain* precipitation (mm)				
Valcartier	0.0	2.2	±	0.3	4.5	0	38	±	4	100	95	306	±	16	873	2.0	5.1	±	0.2	8.2	553	663	±	9	854
Sussex	0.0	2.7	±	0.1	6.5	0	26	±	1	50	12	196	±	6	616	3.9	5.8	±	0.1	7.6	619	745	±	4	1014
Mastigouche	0.0	3.3	±	0.2	7.0	0	44	±	3	100	46	299	±	15	873	1.0	4.2	±	0.2	6.5	553	666	±	9	854

Table S3. Classes of mean sum of aglycon acetophenones (MSAA). These classes result from natural break (Jenks) option of quantitative representation from ArcGIS v10.3 (ESRI, Redlands, CA) analyses that were done for each of the three common gardens in Figure 3. Intervals for each class are exclusive at 10^{-5} mg g^{-1} precision (not shown).

	Natural breaks classes								
	Low			Moderate			High		
Valcartier	0.0	-	6.9	6.9	-	73.6	73.6	-	122.1
Sussex	0.0	-	4.4	4.4	-	12.9	12.9	-	27.9
Mastigouche	0.3	-	14.8	14.8	-	28.8	28.8	-	53.2

Table S4. Regression between mean sum of aglycon acetophenones in *Picea glauca* and biotic and abiotic environmental factors. Each column presents the regression coefficient (r^2) and its associated *P*-value in parenthesis. All variables except mean annual temperature were log transformed.

Common gardens	Historical damage		Forest type		Altitude		Mean annual temperature		Mean annual rain precipitation	
Valcartier	0.01	(0.70)	0.01	(0.59)	0.00	(0.80)	0.12	(0.07)	0.01	(0.71)
Mastigouche	0.01	(0.44)	0.02	(0.35)	0.00	(0.92)	0.09	(0.05)	0.01	(0.55)
Sussex	0.26	(0.005)	0.15	(0.02)	0.02	(0.48)	0.15	(0.02)	0.07	(0.13)

References

1. Mageroy, M. H. *et al.* Expression of the β-glucosidase gene *Pgβglu-1* underpins natural resistance of white spruce against spruce budworm. *Plant J.* **81,** 68–80 (2015).
